# Exploring PEGylated and immobilized laccases for catechol polymerization

**DOI:** 10.1186/s13568-018-0665-5

**Published:** 2018-08-22

**Authors:** Jing Su, Jennifer Noro, Jiajia Fu, Qiang Wang, Carla Silva, Artur Cavaco-Paulo

**Affiliations:** 1 International Joint Research Laboratory for Textile and Fiber Bioprocesses, Jiangnan University, Wuxi, 214122 China; 2 Centre of Biological Engineering, University of Minho, Campus de Gualtar, 4710-057 Braga, Portugal

**Keywords:** Laccase, Polyethylene glycol, Immobilization, PEGylation, Polymerization

## Abstract

Laccases have been reported for their ability to eliminate hazardous phenolic compounds by oxidative polymerization. The exploitation of the oxidative behavior of different laccase forms, namely free/native, free/PEGylated, immobilized/native and immobilized/PEGylated, was assessed in this study. We found that PEGylated and immobilized laccase forms have differentiated catalytic behavior revealing distinct conversion rates and differentiated poly(catechol) chains, as confirmed by UV–Visible spectroscopy, by the total content of OH groups and by MALDI-TOF spectroscopy. The synergy underlying on the immobilized/PEGylated enzyme forms reveal to be responsible for the highest conversion rates and for the longer polymers produced.

**Electronic supplementary material:**

The online version of this article (10.1186/s13568-018-0665-5) contains supplementary material, which is available to authorized users.

## Introduction

Laccases are multicopper proteins considered as one of the most promiscuous enzymes since they can catalyze a broad spectrum of aromatic compounds and their derivatives (Strong and Claus 2011). This makes laccases promising biocatalysts for applications in biotechnological processes, including the detoxification of industrial effluents, textile and petrochemical industries, polymer synthesis, bioremediation of contaminated soils, wine and beverage stabilization, medicine and cosmetic ingredients (Kunamneni et al. 2008). One of the potential applications is on the removal of environmental and industrial pollutants, like phenolic compounds (Rodríguez Couto and Toca Herrera 2006; Viswanath et al. 2014). Several studies have been reporting the polymerization of catechol highlighting different aspects related with the enzymatic catalysis like enzyme performance, degree of polymerization, conversion rate, among others. A study conducted by Gavrillas et al. reported the catechol biotransformation into high molecular species with purified laccase in contrast with the low molecular weight species obtained with a commercial enzyme (Gavrilaş et al. 2012). Aktaş and co-workers studied the kinetics of laccase polymerization reaction and confirmed that a general enzyme kinetics saturation response was observed for catechol substrate during their oxidation, which is probably due to the reduced laccase stability or to a drop in the dissolved oxygen concentration (Aktaş and Tanyolaç 2003b). Although there is a great commercial potential for laccase applications, its instability in harsh conditions is considered a huge issue and strategies need to be explored to stabilize laccase either by chemical modification, immobilization or other techniques (Forde et al. 2010; Shin-ya et al. 2005). Moreover, the elimination of the harsh solvents generally applied is also a concern related with the enzymatic oxidation reactions involving these types of compounds. Several studies have highlighted the identification of reaction products generated by the degradation of phenolic contaminants in water by laccases, however lacking information about the NMR of the final structures obtained (Asadgol et al. 2014; Catherine et al. 2016; Majeau et al. 2010; Shin-ya et al. 2005).

In previous works we, and others, have assessed the effect of PEG on the polymerization of catechol by laccase from *Myceliophthora Thermophila* (Mayolo-Deloisa et al. 2015; Su et al. 2017, 2018). We have found that the previous PEGylation of the enzyme lead to a threefold polymerization activity increase while the single addition of PEG (0.5 mg/mL) to the medium increased only 1.5-fold, when compared with native enzyme. Experiments in a porous acrylamide gel (Ursoiu et al. 2012), where all the catalysts were “frozen”, show no effect of PEG on laccase polymerase activity (Su et al. 2017). The presence of PEG (López-Cruz et al. 2006; Otsuka et al. 2012) increased the average DP by one unit (from 7 to 8) and it is believed that a good mixing between PEG and poly(catechol) enhanced the reaction, as suggested by Molecular Dynamic Studies.

**Fig. 1:**
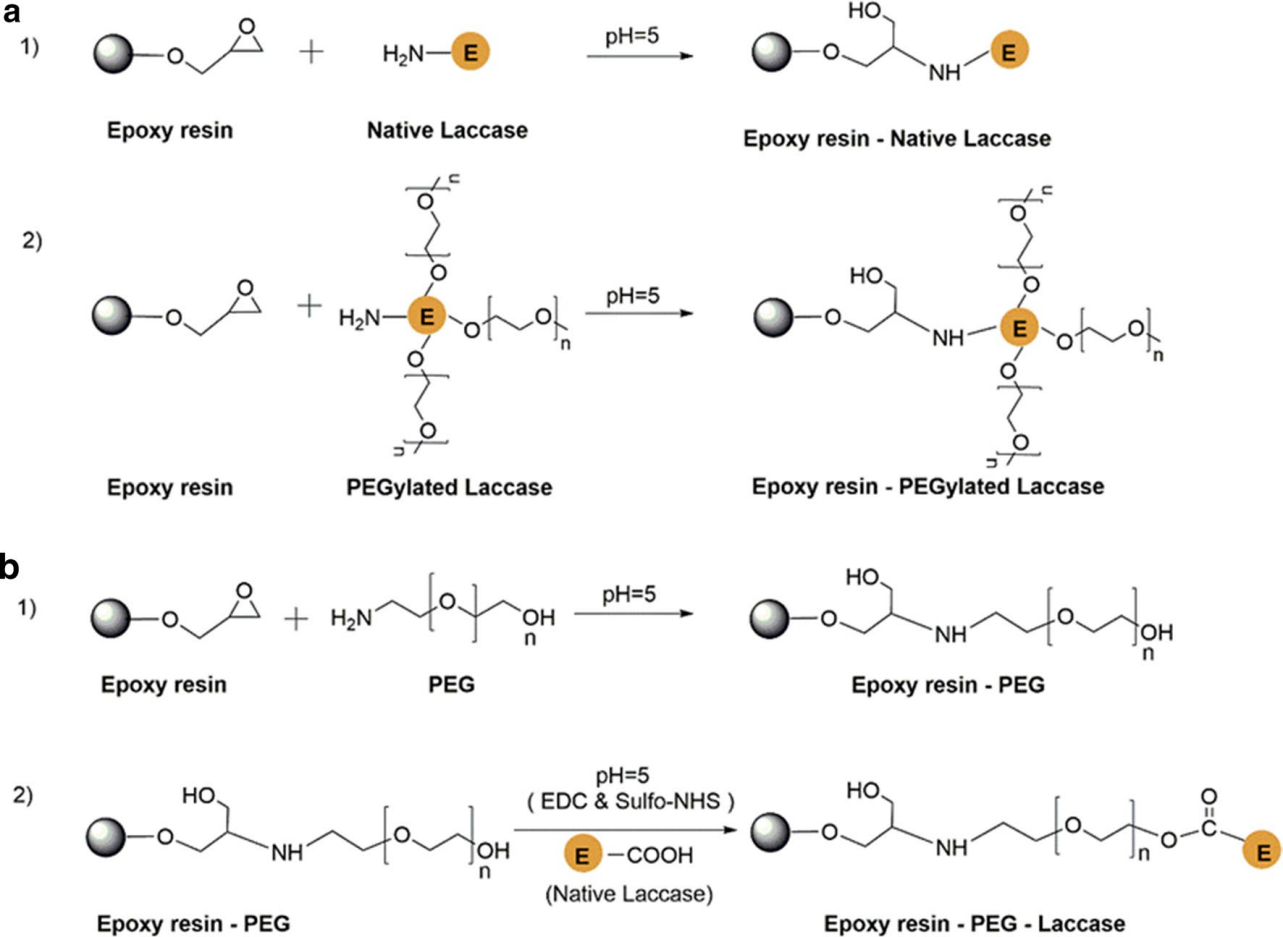
Reactional schemes proposed for the immobilization of laccase onto epoxy resins: **a** covalent immobilization of native laccase (**a**1) and laccase PEGylated (**a**2); **b**1 activation of epoxy resin with (2-aminoethyl) polyethylene glycol (3 kDa); **b**2 covalent immobilization of native laccase onto PEG-activated resin via EDC/sulfo NHS method

Herein, we aim to study the role of both chemical PEGylation and immobilization on the polymerase activity of laccase. The chemical PEGylation was performed by following the methodology described by Daly et al. (2005). The Native and PEGylated laccase forms were immobilized onto epoxy resin according to the method previously described by Berrio et al. (2007) (see reactions in Fig. 1). The epoxy resins were chosen as supports due to their well-known chemistry described in literature (Brady and Jordaan 2009; Chen et al. 2008; Fernández-Fernández et al. 2013; Mateo et al. 2000). Reported data show considerable enhancement of laccases stability when silanized and glutaraldehyde-activated silica nanoparticles are used as supports (Liu et al. 2008). Jimmy and co-workers studied the immobilization of laccase onto epoxy resins-Eupergit C (Berrio et al. 2007) and a substantial stabilization effect against pH and temperature was observed upon immobilization.

The polymerization of catechol was conducted using: (a) native laccase immobilized onto epoxy resin and (b) PEGylated laccase immobilized onto epoxy resin and (c) native laccase immobilized onto PEG-activated resin. Control assays comprising the oxidation of catechol namely with free/native laccase and free/PEGylated laccase, were also performed. UV–Visible spectroscopy was conducted to follow the color change during polymerization. The precipitated polymers were washed with water and methanol to separate the enzyme and the unreacted monomer from the oligomers. The different powder fractions obtained after each washing step were characterized by the Total content of free OH groups (TCFOH), nuclear magnetic resonance spectroscopy (^1^H NMR) and matrix-assisted laser desorption/ionization-time of flight (MALDI-TOF) spectrometry.

## Materials and methods

### PEGylation of laccase

Laccase from *M. thermophila* (supplied by Novozymes, Denmark) was PEGylated as previously reported (Su et al. 2017) using the procedure reported by Daly et al.(2005). Briefly, 14.0 mL of 12 mg/mL laccase were reacted with 20 kDa, *O*-[2-(6-Oxocaproylamino) ethyl]-Omethylpolyethylene glycol at pH 5, 10 mM sodium phosphate buffer with 20 mM sodium cyanoborohydride. A control reaction without mPEG was also conducted in every experiment. The reactions were stirred rapidly for 17 h at 4 °C. After 10 min of mixing, the reagents were completely dissolved, and an aliquot (namely time 0 h) was taken. These samples were ultrafiltrated using a 30 kDa cellulose membrane mounted in an ultrafiltration apparatus. The PEGylated enzyme was then freeze-dried and analyzed by SDS-PAGE electrophoresis.

### Immobilization of native and laccase PEGylated onto epoxy resin supports

The immobilization of native and laccase PEGylated onto epoxy methacrylate resins (Purelite Lifetech ECR enzyme immobilization resins: 300–600 Å) was conducted as follows: 2 mg/mL laccase (native or PEGylated) in 0.5 M acetate buffer (pH 5.0) were mixed with epoxy methacrylate (50 mg/mL) and then stirred for 48 h at 4 °C. The powder was then washed several times with water by centrifugation and dried under vacuum (Berrio et al. 2007).

### Immobilization of native laccase onto PEG-activated epoxy resin supports

Firstly, PEG (*O*-(2-aminoethyl) polyethylene glycol): 3 kDa was used to activate epoxy by nucleophilic attack, as follows: 50 mg/mL PEG in 0.5 M acetate buffer (pH 5.0) was add to epoxy (50 mg/mL) and stirred for 48 h at 4 °C. The powder was washed with water by centrifugation and dried under vacuum. Afterwards, to 5 mg/mL laccase prepared in 0.1 M MES solution (100 mL), were add 40 mg of EDC and 110 mg sulfo-NHS (*N*-hydroxysuccinimide) and mixed for 15 min using orbital agitation at room temperature. The reaction was stopped with 140 µL 2-mercaptoethanol, and the pH adjusted to 7 with PBS (Phosphate-buffered Saline) solution (Bartczak and Kanaras 2011). The final step consisted to add the previously PEG-activated epoxy (10 mg/mL) into the mixed solution and let react for 2 h at room temperature with agitation. Finally, the samples were centrifuged with amicon tubes (100 kDa) to separate the unbound PEG and the free laccase.

### Enzyme stability and half-life time of all laccase forms

The effect of temperature on the enzyme activity and stability of laccase was studied. For this the different forms of laccase were incubated with acetate buffer (pH 5) at 40, 50 and 60 °C, for 150 h. The activity of laccase was measured against ABTS according to the methodology described by Childs and Bardsley (1975). The half-life time of the different forms of laccase was calculated according to the Equation (Zille et al. 2003):

$${\text{Half-life time}} \left( {\hbox{t}}_{\frac{1}{2}} \right) = {\frac{{\text{ln2}}}{{\text{K}}}},\quad {\text{where K}} = \left({\text{ln U}}_{0} - {\text{ln U}}_{\hbox{t}} \right) / {\text{t}},  $$

U_0_ is the enzyme activity at time zero; U_t_ is the enzyme activity at time t; t is the time of incubation, U is the 1 U is defined as the amount of enzyme that catalyzes the conversion of 1 µmol of substrate (ABTS) per minute.

### Enzymatic-assisted polymerization of catechol by free laccase forms

Catechol polymerization was processed by incubating 5 mg/mL of monomer in different solutions: (a) 100 U/mL native laccase, (b) 100 U/mL PEGylated laccase, in acetate buffer (pH 5) (normalized concentration). The reactions were performed in a water bath at 40 °C for 8 h. Afterwards the powder was washed with different solvents, namely five water washings and two methanol washings, under centrifugation, to separate the maximum amount of protein and the non-reacted catechol from the small and big oligomers. Further all the powder fractions recovered from water and methanol extraction step were freeze-dried for posterior analysis. The polymerization reactions were followed during time by UV–Visible spectrometry using the same dilutions for all the solutions obtained.

### Enzymatic-assisted polymerization of catechol by immobilized laccase forms

5 mg/mL of catechol were incubated with: Epoxy-native laccase, Epoxy-laccase PEGylated and Epoxy-PEG-laccase, separately. As controls, free/native laccase and immobilized/native were used to polymerize catechol using the same conditions. The reactions were performed in a water bath at 40 °C for 8 h using 100 U/mL enzyme. Afterwards the polymers were washed with water to remove the protein and separate the oligomers formed from the epoxy by centrifugation with amicon 10 kDa followed by freeze-drying. Then the freeze-dried powder was washed with different solvents, namely five water washings and two methanol washings, under centrifugation, to separate the maximum amount of protein and the non-reacted catechol from the small and big oligomers. Further all the powder fractions recovered from water and methanol extraction step were freeze-dried for posterior analysis. The polymerization reactions were followed during time by UV–Visible spectrometry using the same dilutions for all the solutions obtained.

### Mass spectra analysis

The new polymers were analyzed by matrix-assisted laser desorption/ionization with time-of-flight (MALDI-TOF) using 2,5-dihydroxy benzoic acid (DHB) as the matrix (≥ 99.5%). The mass spectra were acquired on an Ultra-flex MALDI-TOF mass spectrophotometer (Bruker Daltonics GmbH) equipped with a 337 nm nitrogen laser. For this, the samples were dissolved in a TA30 (30% acetonitrile/70% trifluoroacetic acid) solution and mixed with a 20 mg/mL solution of DHB (1:1). Then a volume of 2 μL was placed in the ground steel plate (Bruker part no 209519) until dry. The mass spectra were acquired in analyzed using in linear positive mode.

## ^1^H NMR spectra

The precipitates obtained after washing with water and methanol under centrifugation were dissolved in a deuterated solvent, DMSO-d_6_, for ^1^H NMR evaluation. The amount of OH groups on the samples was evaluated by ^1^H NMR spectra after addition of two water drops to the previous samples in DMSO-d_6_. The spectra were acquired in a Bruker Advance III 400 (400 MHz) using the peak solvent as internal reference.

### Determination of the total content of free OH groups

The total content of free OH groups before and after polymerization was performed using the Folin–Ciocalteu spectrophotometric method. The monomer and polymer solutions dissolved in DMSO (100 µL) were added to the mixture of Folin–Ciocalteu reagent (500 µL) and distilled water (6 mL), and the mixture was shaken for 1 min. Then Na_2_CO_3_ solution (15 wt%, 2 mL) was added to the mixture and shaken for 1 min. Later the solution was brought up to 10 mL by adding distilled water. After 2 h, the absorbance at 750 nm (25 °C) was measured. The total content of free OH was assessed by plotting a gallic acid calibration curve (from 1 to 1500 µg/mL). The equation of the gallic acid calibration curve was A = 0.2977c + 0.0368, and the correlation coefficient was r^2^ = 0.9988.

## Results

### Reaction turnover and amount of poly(catechol) formed

**Fig. 2:**
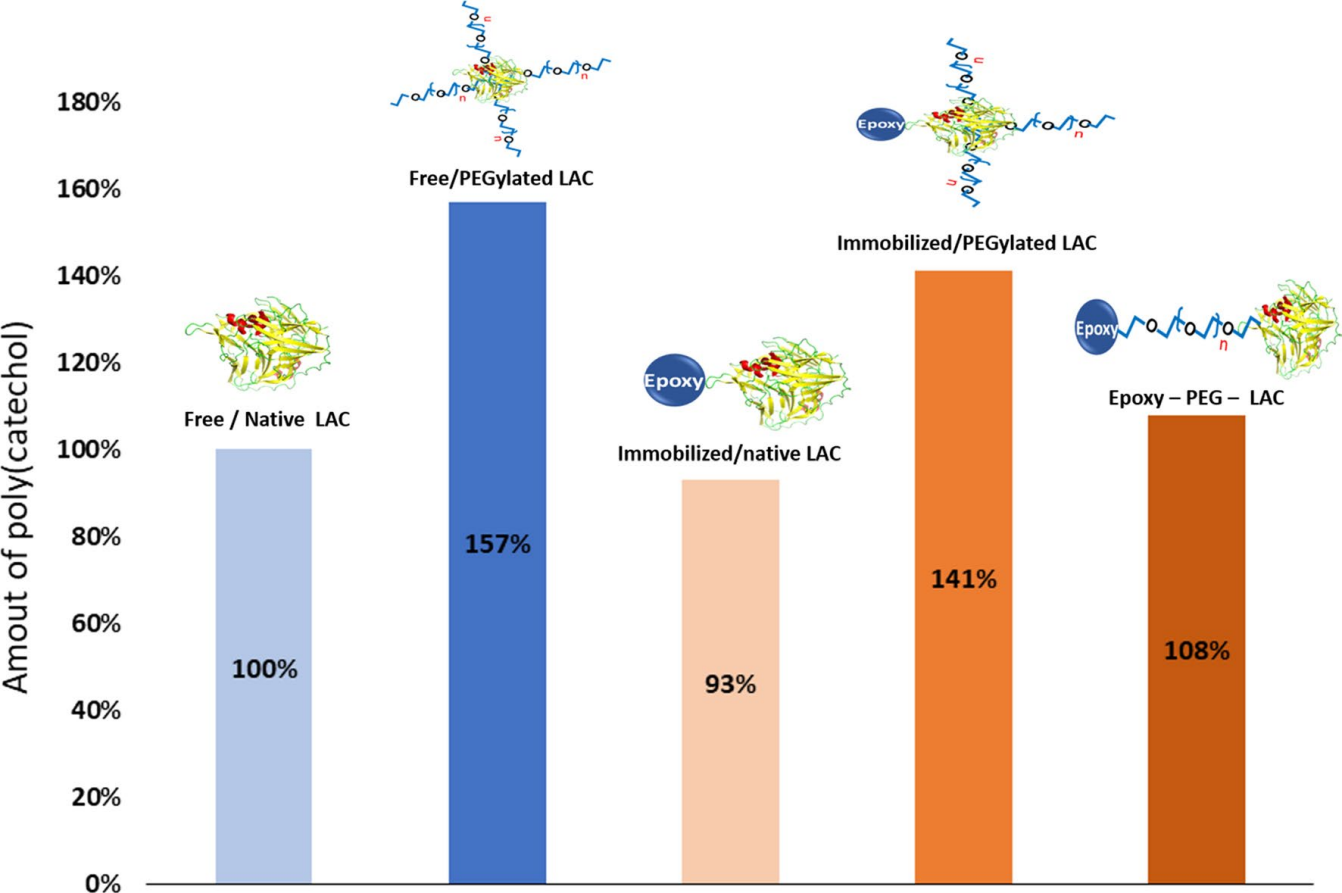
Amount of poly(catechol) after catalysis with: free/native LAC, free/PEGylated LAC, immobilized/native LAC, immobilized/PEGylated LAC and immobilized onto epoxy activated/native; the polymerization was conducted for 8 h at 40 °C using 100 U/mL_enzyme_ (λ = 400 nm; free/native laccase corresponds to 100%) (% of polymer produced was calculated by UV measurements)

The amount of poly(catechol) produced after catalysis with the different forms of laccase, namely free/native LAC, free/PEGylated LAC, Epoxy/native LAC and Epoxy/PEGylated LAC, was evaluated by means of UV/visible spectroscopy. The results reveal that the presence of PEG greatly enhanced the amount of polymer produced (Modaressi et al. 2005; Shoda et al. 2016), either in solution (data from our previous work) or when linked to the enzyme (1.5-fold of increase) (Fig. 2). The catalysis of poly(catechol) with immobilized PEGylated laccase was also enhanced relatively to the immobilized native form. Comparing the catalysis with both, free/native and immobilized/native laccase forms, one can observe a slight decrease of the polymer amount. As expected, the catalytic activity decreases after immobilization due to lower mobility of the catalysts which has some molecular space occupied by the epoxy support.

It is noteworthy that the stability of native and chemically modified laccases was evaluated, and the results confirm the loss of activity of all the enzyme forms particularly pronounced at 60 °C. Free/PEGylated LAC and immobilized/PEGylated LAC present a higher stability than free/native enzyme confirming the stabilization role of both PEG and of the immobilization step (see Additional file 1: Table S1).

### The role of laccase PEGylation and immobilization on catechol polymerization

After polymerization our goal was to perceive if we were in the presence of size-differentiated polymers and in which extent the PEGylation of the catalysts would influence the conversion rate of reaction and the final polymerization degrees.

From the data obtained one can infer that the amount of polymer produced is not the solely alteration observed when the chemically modified and immobilized enzymes were applied (Table 1). The nature of the polymer formed is quite different in size when PEGylated LAC is applied but is more evident when the PEGylated catalyst is in the immobilized form. The average DP increases from 7 to 8 when native/PEGylated is used but increases more considerably when the immobilized laccase form is used (DP = 7 versus DP = 10, comparing free and immobilized native laccase; DP = 8 versus DP = 14, comparing free and immobilized PEGylated laccase).

**Table 1:** Polymerization of catechol under different experimental conditions (laccase amount; OD at 400 nm; total content of free OH, calculated by Folin–Ciocalteu method and by ^1^H NMR; average degree of polymerization and dispersity)

	Activity (U/mL)	OD—8 h (400 nm)	Amount of OH groups		(Mn; Mw)^a^	Average DP^a^	*D*_*M*_ (dispersity)
			By Folin–Ciocalteu method	By ^1^H NMR			
Free/native LAC	100	0.926	0.515	0.500	748; 776	7	1.03
Free/PEGylated LAC		1.452	0.293	0.310	833; 849	8	1.02
Immobilized/native LAC		0.857	0.192	–	1088; 1118	10	1.03
Immobilized/PEGylated LAC		1.301	0.202	0.110	1460; 1498	14	1.02
Epoxy-PEG-LAC		1.003	0.075	0.320	1570; 1600	15	1.02

^a^Average degree of polymerization calculated by MALDI-TOF analysis; 1 U is defined as the amount of enzyme that catalyzes the conversion of 1 µmol of substrate (ABTS) per minute per mg of protein used

**Fig. 3:**
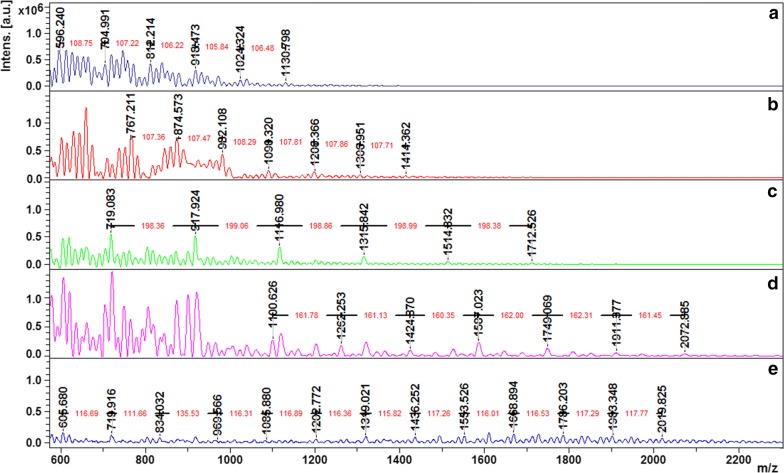
MALDI-TOF mass spectra of poly(catechol) catalyzed by: (a) free/native LAC; (b) free/PEGylated LAC; (c) immobilized/native LAC; (d) immobilized/PEGylated and (e) native immobilized onto PEG-activated resin

The MALDI-TOF data (Fig. 3 and Table 1) also reveal that the biocatalysis using the solid support lead to the formation of poly(catechol) with a higher polymerization degree (DP = 10 versus DP = 7) comparing both free/native and immobilized/native enzyme. The immobilized/PEGylated form present higher DP than free/PEGylated one (DP = 14).

The determination of the total content of free OH groups by different methodologies, namely Folin–Ciocalteu (Blainski et al. 2013; Singleton et al. 1999) and ^1^H NMR methods allowed us to distinguish different catalytic behaviour between all the laccase forms used for catechol polymerization. Comparing both native and PEGylated laccase forms, the data obtained show a significant decrease of the total content of OH groups for the modified form, even on free or in the immobilized form (see Additional file 2: Figure S1).

By ^1^H NMR spectra analysis one can perceive broad signals observed between δ_H_ 6.0 and 8.5 ppm, corresponding to the aromatic peaks of poly(catechol). This indicates that we are in the presence of complex mixtures after polymerization with all enzyme forms (Additional file 2: Figure S1). Due to spectra complexity no polymer structure can be elucidated. Still, another broad signal can be detected between δ_H_ 8.5 and 10.5 ppm, which corresponds to OH signal group. This assumption was confirmed by D_2_O addition to the NMR tube (data not shown). The OH content contained in all the reaction samples was calculated by ^1^H NMR integration and are presented in Table 1.

**Fig. 4:**
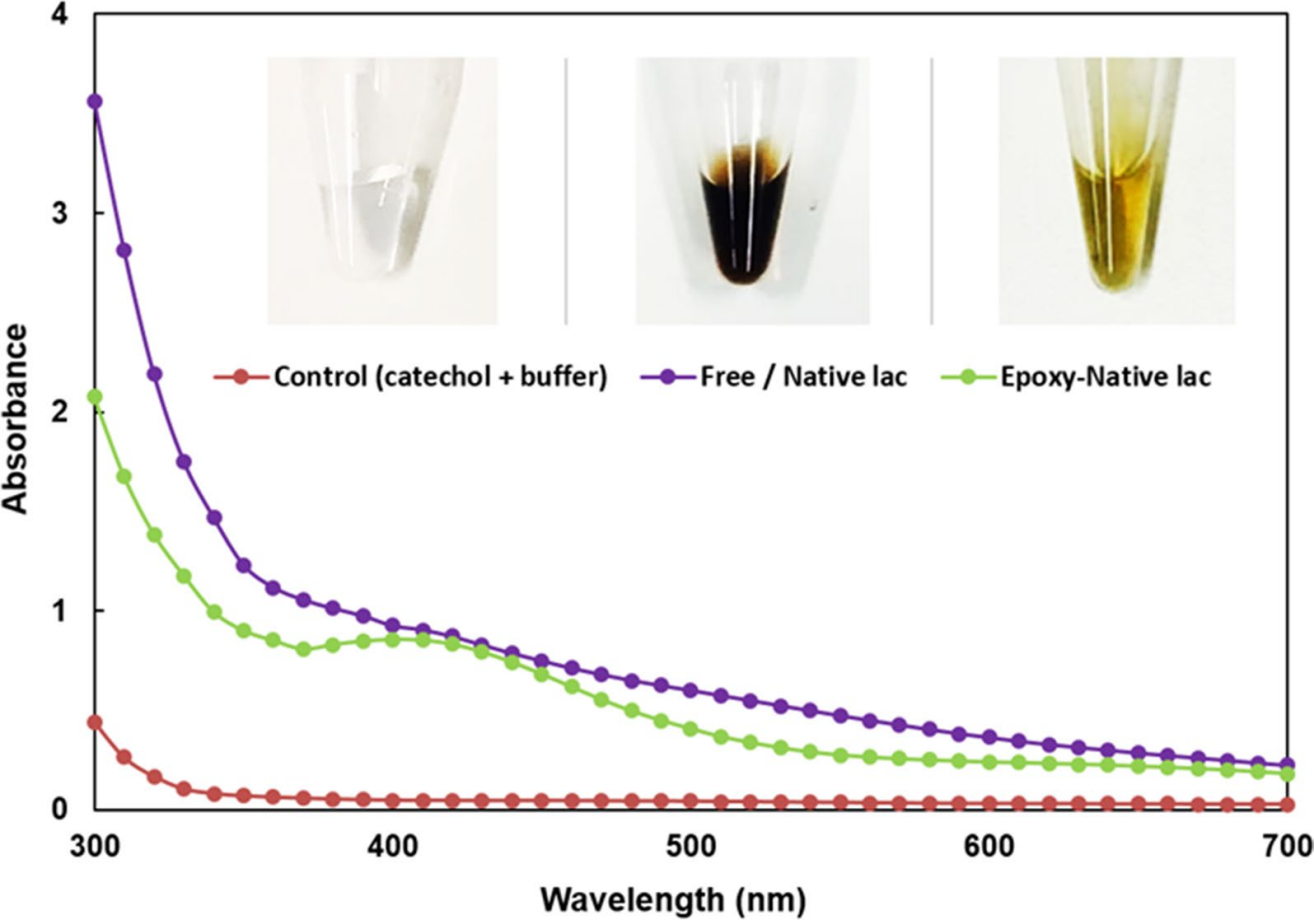
UV–Visible spectra of poly(catechol) after polymerization using: control (buffer + catechol) (red line), free/native laccase (purple line) and immobilized/native laccase (green line); the polymerization was performed using 100 U/mL_enzyme_ for 8 h at 40 °C

The immobilization of the catalysts, in the native or PEGylated form, influenced greatly the laccase performance as demonstrated in Fig. 4. Free/native LAC in solution conditions yields polymers with the typical dark brown color presenting a typical UV/Vis spectrum (Fig. 4, in purple). When immobilized, the enzymes produce green–brown polymers with a different spectra behavior (Fig. 4, in green).

## Discussion

The catalytic ability of different laccase forms (free/native LAC, free/PEGylated LAC, immobilized/PEGylated LAC and Epoxy-PEG-LAC) to polymerize catechol was evaluated. Overall the data from Table 1 and Fig. 2, considering the amount of poly(catechol) formed and the polymerization degrees obtained, indicates that PEGylated laccase, free or immobilized onto the epoxy resin, seems to be the ideal catalyst for catechol polymerization. Previously Modaressi and co-workers described the protective role of PEG on laccase activity (Modaressi et al. 2005). Herein, we confirm also that the presence of PEG linked to the enzyme greatly enhanced the amount of polymer produced, as set by UV–Visible spectroscopy. The color change of the catechol solution is associated with a bathochromic shift and an increase in the UV–Vis absorption intensity indicates a greater degree of π-conjugation thus confirming polymerization (Jha and Halada 2011). The results demonstrate other potentiality of PEG, which can not only be used as stabilizer, in solution or linked to the enzyme, but also as a spacer/linker for the immobilization of laccase onto solid supports (López-Cruz et al. 2006).

The free hydroxyl content and MALDI-TOF data (Table 1, Fig. 3), the UV/visible spectra (Fig. 4) indicate that, independently on the amount of poly(catechol) produced, the different laccase forms used yield size-different polymers. The PEGylation and immobilization of the catalyst influence greatly the catalytic behavior of laccase. The role of PEG as template for polymerization reactions is not new, some works have already reported the role of this additive as template to assist polymerization reactions (Modaressi et al. 2005; Su et al. 2017; Wu et al. 1997). Previously we also confirmed that the oxidation of phenolics like catechol is favored when conducted in the presence of PEG, even in solution or linked to the enzyme. The PEGylation of laccase enhanced the polymerase activity by threefold compared with the native enzyme, while PEG in solution only increased polymerization by 1.5-fold. Our results were corroborated by molecular dynamics simulations which suggested the formation of a miscible complex between PEG and the poly(catechol) formed, pushing the reaction forward. Herein, our data also confirms these previous findings, showing higher conversion rates and longer polymers when PEGylated enzyme is applied. Moreover, the only report about the effect of PEGylated MtL on other substrates (López-Cruz et al. 2006) revealed that K_cat_ and K_m_ are not significantly affected by PEGylation, confirming our previous assumptions.

Several reports highlight the conformational changes suffered by enzymes when immobilized which may alter their catalytic properties (Brady and Jordaan 2009; Fernández-Fernández et al. 2013; Halling et al. 2005). However, it is also established that when immobilized, the enzymes became stable and less susceptible to the medium interference like obstruction of the catalytic active site by the new polymers formed. Our data indicates that immobilized laccase gave rise to a higher decrease of the free hydroxyls, indicating the formation of a longer polymer. From UV/Visible spectra data one can predict a synergistic effect between PEGylation and immobilization, since immobilized/native enzyme did not reveal higher performance when compared with free/native catalyst. On the other hand, when PEGylated laccase form, or even native enzyme is immobilized onto PEG activated resin, the conversion increases and longer polymers are obtained. The immobilization of the catalyst together with the presence of the PEG are expected to stabilize the enzyme allowing the reactions to proceed longer with the formation of polymers with higher polymerization degrees. Comparing with several works reported in literature, the modified laccase forms herein presented are promising catalysts to efficiently produce longer polymers (Aktaş and Tanyolaç 2003a; Sun et al. 2013).

The literature related with the enzymatic polymerization of catechol report a typical polymer structure where the catechol units are connected by ether linkages. The reaction occurs by oxygen-carbon bonding at the *para*-position of the other monomeric unit. This position is more prone to be involved in the reaction binding rather than the *orto*-position, which involves more stereochemical impediments (Aktaş and Tanyolaç 2003; Aktaş et al. 2003; Liu et al. 2015; Sun et al. 2013; Zerva et al. 2016). Both formation of quinoid derivatives or homomolecular dimers coming from an intermolecular nucleophilic attack from the radicals formed by reaction with laccases have been described. The units composing these dimers are linked by C–C or C–O bonds by oxidative condensation, oxidative phenolic coupling or oxidative coupling. After a certain reaction time, this coupling can lead to the formation of oligomers or polymers (Catherine et al. 2016).

The complexity to identify these oligomers as well as to establish the corresponding polymerization reaction pathways, especially when different laccase forms are applied, is still a major drawback to overcome. The structure identification of some polymeric products is not completely clear and need deeper investigations. Moreover, despite the high number of works reporting the enzymatic polymerization of catechol, no elucidation about the polymer NMR is presented, neither about the fractionation of the final polymers obtained. The authors generally base their structural proposals on the previous findings reported previously. Contrarily to what is reported, after enzyme and unreacted catechol removal, we were not able to predict a possible polymer structure. The ^1^H NMR and C-13 NMR (not shown) reveal a very broad spectra which does not allow the correct prediction of the possible polymer structures. Even though, the structure generally assigned in literature as being the final poly(catechol) is also not identified in all the spectra acquired after catalysis with all enzyme forms. From all the spectra obtained one can infer that we are in the presence of a panoply of different polymer structures with differentiated lengths, however impossible to identify.

## Additional files

**Additional file 1: Table S1.** Half-life time of enzymes *vs* temperature of incubation.

**Additional file 2: Figure S1.** ^1^H NMR of powder fraction polymerized by **a)** free/native laccase; **b)** free/PEGylated; **c)** immobilized/PEGylated laccase and **d)** native laccase immobilized onto PEG-activated resin, after washings with water and with methanol (in DMSO-d6).

## Abbreviations

PEG: polyethylene glycol
DP: the degree of polymerization
TCFOH: the total content of free OH groups
NMR: nuclear magnetic resonance spectroscopy
MALDI-TOF: matrix-assisted laser desorption/ionization-time of flight
LAC: laccase
EDC: 1-ethyl-3-(3-dimethylaminopropyl) carbodiimide
DHB: 2,5-dihydroxybenzoic acid
DMSO: dimethyl sulfoxide
