# Supplementary material for: Exploring PEGylated and immobilized laccases for catechol polymerization
Source: AMB Express. 2018 Aug 22;8:134. doi: 10.1186/s13568-018-0665-5 (PMC6104406; doi:10.1186/s13568-018-0665-5)
Supplement: Supplementary file 1 — Additional file 1: Table S1. Half-life time of enzymes vs temperature of incubation. [file 13568_2018_665_MOESM1_ESM.docx]

Additional file

AMB Express

**Exploring PEGylated and immobilized laccases for catechol polymerization**

Jing Su^[a,b]^, Jennifer Noro^[b]^, Jiajia Fu^[a]^, Qiang Wang^[a]^, Carla Silva,^[b]^ and Artur Cavaco-Paulo*^[a,b]^

^a^International Joint Research Laboratory for Textile and Fiber Bioprocesses, Jiangnan University, Wuxi 214122, China

^b^Centre of Biological Engineering, University of Minho, Campus de Gualtar, 4710-057, Braga, Portugal

**^#^Corresponding Author**

Artur Cavaco-Paulo; [artur@deb.uminho.pt](mailto:artur@deb.uminho.pt)

Centre of Biological Engineering, University of Minho, Campus de Gualtar, 4710-057, Braga, Portugal;

International Joint Research Laboratory for Textile and Fiber Bioprocesses, Jiangnan University, Wuxi 214122, China;

**Tables**

**Table S1.** Half-life time of enzymes *vs* temperature of incubation

| **Incubation temperature (°C)** | **Half-life time (t_1/2_) (h) ± SD** | | |
| --- | --- | --- | --- |
|  | **Native laccase** | **PEGylated laccase** | **Epoxy-PEGylated laccase** |
| **40** | 41.3±4.2 | 48.4±4.3 | 46.9±4.5 |
| **50** | 21.1±3.5 | 26.2±3.4 | 26.7±4.1 |
| **60** | 12.9±2.9 | 20.4±2.6 | 19.3±3.9 |
