# Supplementary material for: Exploring PEGylated and immobilized laccases for catechol polymerization
Source: AMB Express. 2018 Aug 22;8:134. doi: 10.1186/s13568-018-0665-5 (PMC6104406; doi:10.1186/s13568-018-0665-5)
Supplement: Supplementary file 2 — Additional file 2: Figure S1. 1H NMR of powder fraction polymerized by a) free/native laccase; b) free/PEGylated; c) immobilized/PEGylated laccase and d) native laccase immobilized onto PEG-activated resin, after washings with water and with methanol (in DMSO-d6). [file 13568_2018_665_MOESM2_ESM.docx]

**Figures**

**
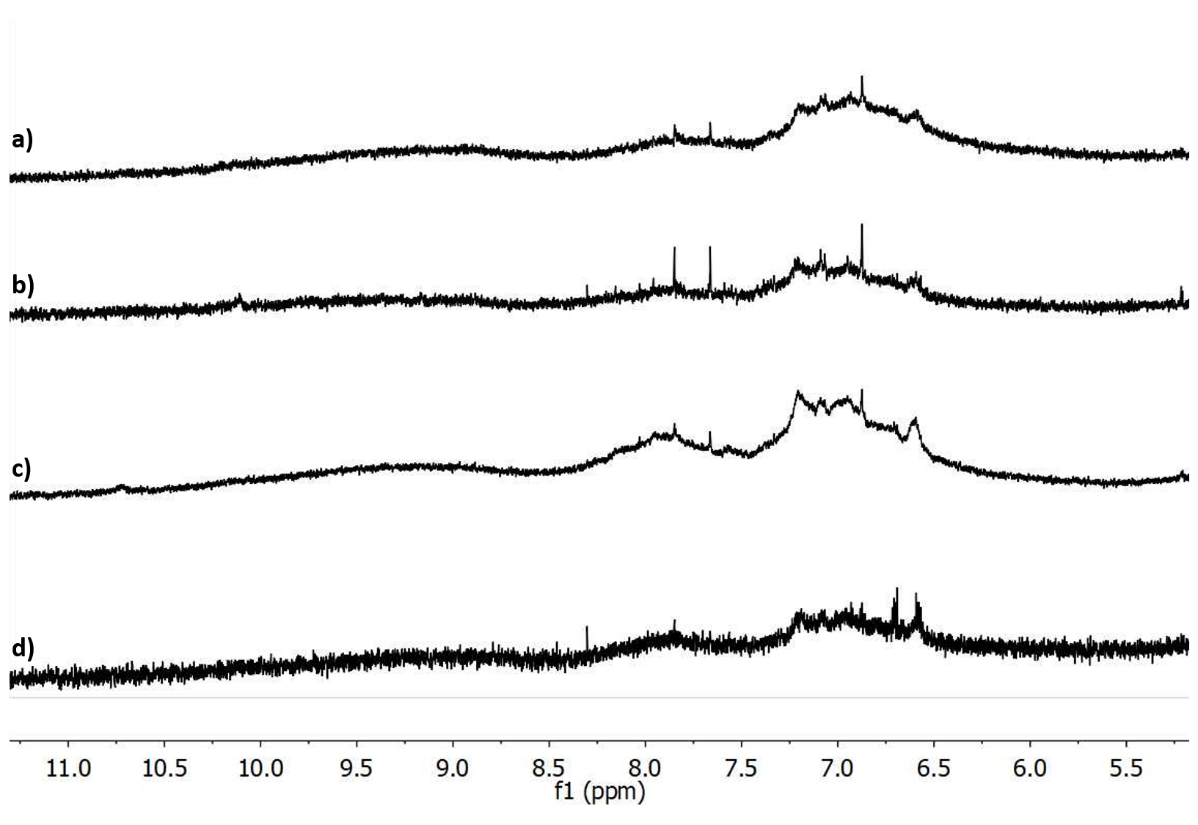
**

**Figure S1:** ^1^H NMR of powder fraction polymerized by **a)** free/native laccase; **b)** free/PEGylated; **c)** immobilized/PEGylated laccase and d) native laccase immobilized onto PEG-activated resin, after washings with water and with methanol (in DMSO-d6).
